# Objective vs. Self-Reported Physical Activity and Sedentary Time: Effects of Measurement Method on Relationships with Risk Biomarkers

**DOI:** 10.1371/journal.pone.0036345

**Published:** 2012-05-09

**Authors:** Carlos A. Celis-Morales, Francisco Perez-Bravo, Luis Ibañez, Carlos Salas, Mark E. S. Bailey, Jason M. R. Gill

**Affiliations:** 1 Institute of Cardiovascular and Medical Sciences, College of Medical, Veterinary and Life Sciences, University of Glasgow, Glasgow, United Kingdom; 2 School of Life Sciences, College of Medical, Veterinary and Life Sciences, University of Glasgow, Glasgow, United Kingdom; 3 Laboratory of Nutritional Genomics, Department of Nutrition, Faculty of Medicine, University of Chile, Santiago, Chile; 4 Center of Nutrition, Metabolism and Physical Activity (CNEF), Concepción, Chile; 5 Department of Physical Education, University of Concepción, Concepción, Chile; McGill University, Canada

## Abstract

**Purpose:**

Imprecise measurement of physical activity variables might attenuate estimates of the beneficial effects of activity on health-related outcomes. We aimed to compare the cardiometabolic risk factor dose-response relationships for physical activity and sedentary behaviour between accelerometer- and questionnaire-based activity measures.

**Methods:**

Physical activity and sedentary behaviour were assessed in 317 adults by 7-day accelerometry and International Physical Activity Questionnaire (IPAQ). Fasting blood was taken to determine insulin, glucose, triglyceride and total, LDL and HDL cholesterol concentrations and homeostasis model-estimated insulin resistance (HOMA_IR_). Waist circumference, BMI, body fat percentage and blood pressure were also measured.

**Results:**

For both accelerometer-derived sedentary time (<100 counts.min^−1^) and IPAQ-reported sitting time significant positive (negative for HDL cholesterol) relationships were observed with all measured risk factors – i.e. increased sedentary behaviour was associated with increased risk (all *p*≤0.01). However, for HOMA_IR_ and insulin the regression coefficients were >50% lower for the IPAQ-reported compared to the accelerometer-derived measure (*p*<0.0001 for both interactions). The relationships for moderate-to-vigorous physical activity (MVPA) and risk factors were less strong than those observed for sedentary behaviours, but significant negative relationships were observed for both accelerometer and IPAQ MVPA measures with glucose, and insulin and HOMA_IR_ values (all *p*<0.05). For accelerometer-derived MVPA only, additional negative relationships were seen with triglyceride, total cholesterol and LDL cholesterol concentrations, BMI, waist circumference and percentage body fat, and a positive relationship was evident with HDL cholesterol (*p* = 0.0002). Regression coefficients for HOMA_IR_, insulin and triglyceride were 43–50% lower for the IPAQ-reported compared to the accelerometer-derived MVPA measure (all *p*≤0.01).

**Conclusion:**

Using the IPAQ to determine sitting time and MVPA reveals some, but not all, relationships between these activity measures and metabolic and vascular disease risk factors. Using this self-report method to quantify activity can therefore underestimate the strength of some relationships with risk factors.

## Introduction

There is clear evidence from a large body of epidemiological data that high levels of physical activity are associated with reduced risk of a number of adverse health outcomes, including type 2 diabetes [1], cardiovascular disease [2], and mortality from any cause [2]. In addition, there is a growing body of evidence that high levels of sedentary time – usually assessed as time spent sitting or watching television – are also associated with adverse metabolic and vascular health outcomes [3]–[7], and these effects are often independent of physical activity level.

However, quantification of the strength and nature of the relationship between physical activity (or sedentary behaviour) and health outcomes in population-based studies is reliant on accurate measurement of activity behaviour: poor methods increase chances of misclassification and can add bias, which can mask or distort the true underlying relationship between activity and health [8], [9]. Much of the evidence generated in this area – on which current physical activity guidelines have largely been based [10], [11] – has derived from estimates of physical activity or sedentary behaviour from self-report questionnaires. Such questionnaires are easy to administer, inexpensive and do not alter behaviour, making them well suited to large-scale investigations [8], [9]. However, activity information derived from self-report is potentially subject to response bias (e.g. imprecise recall, influence of social desirability) [12] – and thus validation of activity questionnaires against criterion measures is vital [13]. One commonly used physical activity questionnaire is the International Physical Activity Questionnaire (IPAQ), which, in its long-form, provides a comprehensive measure of activity in a variety of contexts (occupational, transport, household, leisure) and intensity (sitting, moderate, vigorous, walking, cycling) domains [14]. In validation studies the IPAQ performs at least as well as other self-report activity measures [13], [14]; criterion validity of the long-form IPAQ (last 7-days) has been assessed, with ‘fair to moderate’ associations being reported between measures of total activity derived from IPAQ and criterion accelerometer methods (r-values ranging from 0.05 to 0.55 in different populations) [14], [15]. Reported correlations between IPAQ-derived sitting time and accelerometer-derived sedentary time (<100 counts.min^−1^) have also been relatively modest (r = 0.14 to 0.51) [14]–[16].

Imprecise measurement of activity variables can lead to a diminution of the apparent effects of activity on health-related outcomes due to regression dilution bias [17]. It is therefore conceivable that the apparent effects of physical activity and sedentary behaviour on risk for metabolic and vascular disease are attenuated when the activity variables are assessed using relatively imprecise self-report questionnaires, compared to when activity is assessed by objective techniques such as accelerometry. This has potentially important implications for the determination of the precise dose-response relationships between activity and health benefits, with associated implications for recommendations for the amounts and types of physical activity needed for optimal health. Thus, additional validation to quantify the magnitude of this potential error with questionnaire-based physical activity or sedentary time assessment is important. A recent study, using the NHANES database, has compared the relationship between self-reported and accelerometer-measured moderate-to-vigorous activity (MVPA) on health-related biomarkers, reporting stronger associations for the objective activity measures [18]. However, the self-report questionnaire used in NHANES has not been as extensively validated as the IPAQ [19], and there have been no published data comparing the strength of relationship between self-reported and objectively assessed sedentary behaviour and metabolic and vascular disease risk factors.

The aim of the present study was therefore to determine whether the strength of relationships for physical activity and sedentary behaviour with risk factors for metabolic and vascular disease differed between measures obtained from IPAQ *vs* objective accelerometer in a population of adults with a wide range of physical activity levels living in rural and urban settings.

## Methods

### Ethics statement

All participants gave written informed consent prior to inclusion in this study, which was approved by Research Ethics Committees at the University of Glasgow, University of Chile, and University of Concepción.

### Participants

Participants were 317 adults (140 men, 177 women), aged 18–73 years drawn from a wider study investigating the effects of environment and ethnicity on metabolic risk in the Chilean population [20]. Of these, 163 were of European ethnic origin and 154 were of Mapuche origin (an Amerindian group native to Chile); 163 participants lived an urban environment and 154 lived rurally. Their characteristics are shown in **Table 1** **.** Individuals with a known history of cardiovascular disease or taking anti-hypertensive or diabetes medications were excluded from participation.

**Table 1:** Characteristics of participants.

Variable	Mean ± SD	Range
Age (y)	37.5±12.8	(18–73)
Sex (men/women)	140/177	
Glucose (mmol.l^−1^)	5.52±1.17	(2.75–11.74)
Insulin (mU.ml^−1^)	6.87±12.37	(0.63–78.49)
HOMA_IR_	2.09±2.88	(0.12–18.78)
Total Cholesterol (mmol.l^−1^)	4.75±1.21	(2.61–9.77)
HDL cholesterol (mmol.l^−1^)	0.92±0.38	(0.38–2.31)
LDL cholesterol (mmol.l^−1^)	3.27±1.29	(0.77–8.03)
Triglyceride (mmol.l^−1^)	1.24±0.61	(0.18–3.34)
Systolic BP (mm Hg)	123.2±16.7	(83–181)
Diastolic BP (mm Hg)	76.6±12.4	(39–118)
Body Mass Index (m.kg^−2^)	29.2±5.1	(18.4–49.3)
Waist Circumference (cm)	102.3±13.7	(74.5–136.0)
Body Fat (%)	30.3±5.9	(14.4–47.5)

### Measurement of physical activity by accelerometer

Participants wore accelerometers (ActiTrainer, ActiGraph, LLC, Pensacola, FL, USA) on the left hip at all times, except when showering, swimming and sleeping, for seven consecutive days to objectively assess physical activity levels. Accelerometer readings were summarized in 60-second epochs and Freedson cut-points used to define intensity domains (light <1952 count.min^−1^; moderate 1952–5724 count.min^−1^; vigorous >5725 count.min^−1^) [21]. Data from participants with at least 10 hours of daily accelerometer wear time for 4 days were included in the analysis. Non-wear was defined by intervals of at least 60 minutes of zero activity counts [22]. Wear time was calculated by subtracting non-wear time from 24 hours. Activity count values of <100 count.min^−1^ were defined as sedentary behaviour [23]. Activity was reported as minutes per day of sedentary time, moderate activity and vigorous activity and as MET-minutes (where 1 MET is equivalent to resting energy expenditure) of moderate-to-vigorous physical activity (minutes of moderate activity×4.0 METs+minutes of vigorous activity×8.0 METs). These specific MET values within the moderate (3.0–6.0 METs) and vigorous (>6.0 METs) intensity ranges were chosen for consistency with the MET values assigned for moderate and vigorous activity in the IPAQ scoring protocol (see below).

### Measurement of physical activity by IPAQ

Physical activity and sitting time were measured using a Spanish language long-form, last 7-day, self-administered version IPAQ, which was completed immediately following their 7 days of accelerometer wear [14]. This version of the IPAQ asks questions about the amount of walking undertaken and participation in moderate and vigorous activities in work, transportation, domestic and garden, and leisure domains, amount of cycling undertaken for transport, and time spent sitting on weekdays and weekend days, over the preceding 7 days [14]. Data were analyzed in accordance with the IPAQ scoring protocol (https://sites.google.com/site/theipaq/scoring-protocol). Although walking is a moderate intensity activity by MET value, the IPAQ questionnaire includes walking as a separate activity domain from moderate activity, explicitly excluding walking in the moderate activity questions. Thus, to provide a comparable index to the accelerometer-derived moderate activity measure, the walking and moderate activity domains from the IPAQ were combined into a single ‘moderate’ activity domain for analysis. However, in line with the IPAQ scoring protocol, walking was assigned an intensity of 3.3 METs, with all other moderate activity assigned an intensity of 4.0 METs. Thus, IPAQ data are reported as minutes per day of sitting, moderate activity (including walking) and vigorous activity and as MET-minutes of MVPA (minutes of walking×3.3 METS+minutes of moderate activity (excluding walking)×4.0 METS+minutes of vigorous activity×8.0 METS). Transport cycling activity was not included in analyses, but this is unlikely to substantially influence the study findings as only 13% of the study population reported any cycling for transport and mean reported cycling activity was only 5.0 minutes per day in this cohort.

### Physical, biochemical and demographic measurements

Height, body mass, waist and hip circumferences and skinfolds at four sites (biceps, triceps, subscapular, suprailiac) were measured using standard protocols [24]. Body composition was calculated from skinfold measures [25]. Blood pressure was measured on the right arm after at least 10 minutes of seated rest using an automated blood pressure monitor (Omron HEM705 CP, Omron Healthcare UK Limited, Milton Keynes, UK) which has been validated according to the European Society of Hypertension International Protocol [26]. The mean of two blood pressure readings was used in analysis.

Venous blood samples were drawn after an overnight fast and collected into potassium EDTA tubes and placed on ice. Plasma was separated within 10 minutes of collection and frozen at −20°C until analysis. Glucose, triglyceride (TG), total cholesterol and HDL cholesterol, were determined by enzymatic colorimetric methods using commercially available kits (Roche Diagnostics Gmbh, Mannheim, Germany; Randox Laboratories Ltd., Co. Antrim, Ireland; and Kamiya Biomedical, Seattle, USA). LDL cholesterol was calculated using the Friedewald equation [27]. Insulin concentrations were determined by radioimmunoassay (Diagnostic System Labs, TX, USA). Coefficients of variation were <3.0% for all enzymatic colorimetric assays, 5.0% for insulin.

Socioeconomic status was determined with the European Society for Opinion and Marketing Research (ESOMAR) questionnaire validated in the Chilean population [28]. The original 6 ESOMAR socioeconomic classes were re-grouped into three classes by combining the two lower, two middle and two higher classes for analysis. Demographic and cultural data (age, attained education, most recent occupation, and ethnicity) were determined using the Chilean Socioeconomic Characterisation Questionnaire [29]. All questionnaire data were collected during in-person interviews.

### Data analysis

Data were analysed using STATA (version 11; Statacorp, TX, USA). Accelerometer- and IPAQ-derived values for time or MET-minutes in the different intensity domains were compared by paired *t*-test, and the bias and variability between the two measurement methods for each intensity domain was determined using a limits of agreement approach [30]. The relationships between accelerometer-derived and IPAQ-reported activity measures were assessed using Pearson correlations (r) and concordance correlation coefficients (P_c_). The latter correlation adjusts the r-value using a bias correction factor, which measures how far the best-fit line deviates from the line y = x. P_c_ therefore provides a composite measure of correlation and agreement between the two measures.

The relationships between activity variables and risk factor levels for the accelerometer-derived and IPAQ reported activity measures were determined in two ways. Firstly, to determine the strength of relationships between activity variables and risk factor levels, β values for the unit change in each risk factor with unit (or unit multiple) change in each accelerometer-derived or IPAQ-reported measure of activity were calculated using general linear regression models, adjusted for age, sex, ethnicity, environment (rural or urban), socio-economic status and smoking. To determine whether the strength of relationships differed between the accelerometer and IPAQ activity measures, the analyses were repeated with the equivalent accelerometer and IPAQ measures both included in the same model (adjusted for the same confounders as above) and the interaction between the accelerometer- *vs* IPAQ-derived regression lines was assessed. This, in effect, assesses whether the β values for the effects of accelerometer and IPAQ measures on risk factors differed significantly.

Secondly, to determine whether IPAQ-reported and accelerometer-derived activity measures would similarly detect trends for differences in risk factors across the population distribution of physical activity and sedentary time, participants were divided into quartiles for accelerometer-derived MVPA and sedentary time and for IPAQ-reported MVPA and sitting time. General Linear Models were used to determine effects of increasing MVPA or sedentary/sitting time quartile (included in the model as an ordinal variable) on each risk factor, in models adjusted for age, ethnicity, sex, environment (rural or urban), socio-economic status and smoking status. Models for MVPA were then further adjusted for sitting time (for IPAQ) or sedentary time (for accelerometer) and models for sitting/sedentary time were adjusted for MVPA. To determine whether the relationships between MVPA or sedentary/sitting time quartile and risk factors differed between the accelerometer and IPAQ activity measures, the analyses were repeated with the equivalent accelerometer and IPAQ measures for MVPA or sedentary/sitting time both included in the same model (adjusted for the same confounders as above) and the interaction between the accelerometer-derived *vs* IPAQ-reported relationships were assessed.

Statistical significance was accepted at *p*<0.05.

## Results

### Agreement and correlation between accelerometer-derived and IPAQ-reported activity measures

**Figure 1:**
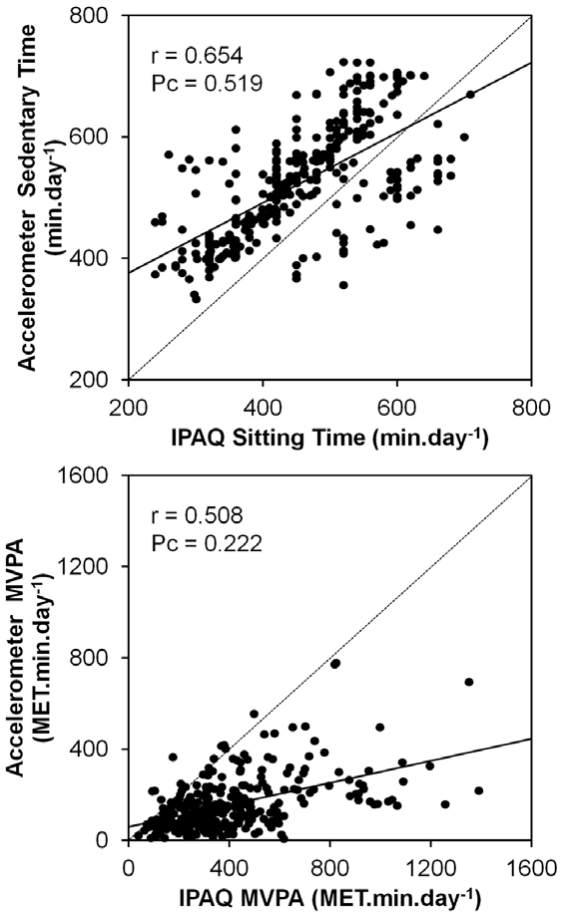
Relationships between accelerometer-derived sedentary time and IPAQ-reported sitting time (top panel) and between accelerometer-derived and IPAQ-reported MVPA (bottom panel). Solid line on each plot represents the linear regression line; dotted line represents the line of equality, y = x. Pearson (r) and concordance (P_c_) correlation coefficients shown.

Participants had a wide range of physical activity and sedentary behaviour levels (Table 2, Figure 1). Mean IPAQ-reported sitting time was ∼13% lower than accelerometer-derived sedentary time (*p*<0.0001). Both the Pearson and concordance correlation coefficients between these two indices of sedentary behaviour were reasonably strong (Figure 1). In contrast, agreement between accelerometer-derived and IPAQ-reported measures of physical activity behaviour was much poorer. IPAQ-reported estimates of moderate activity, vigorous activity and MVPA were 2.6-, 4.0- and 2.6-fold higher, respectively, than the corresponding accelerometer-derived measures of these indices (all *p*<0.0001). Pearson correlations between accelerometer-derived and IPAQ-reported indices of moderate activity and of MVPA were reasonably strong, but the Pearson correlation between the two vigorous activity measures was modest (Table 2). Concordance correlation coefficients for moderate activity, vigorous activity and MVPA were all weak (P_c_≤0.22), reflecting the large divergence of the regression lines for these correlations from the line of equality (Figure 1 illustrates these data for MVPA).

**Table 2:** Accelerometer-derived and IPAQ-estimates indices of physical activity and sedentary behaviour.

Accelerometer-derived *vs* IPAQ-reported activity measure	Accelerometer	IPAQ	Difference [IPAQ minus accelerometer]	*p* b	r (95% CI)	P_c_ (95% CI)
	(Mean ± SD)	(Mean ± SD)	(Mean (Limits of Agreement))			
Sedentary *vs.* Sitting (min.day^−1^)	523.1±90.9	454.2±103.1	−68.8 (−228.3 to 90.8)	<0.0001	0.654 (0.570 to 0.738)	0.519 (0.402 to 0.697)
Moderate *vs.* Moderate^a^ (min.day^−1^)	33.7±23.7	88.5±63.1	54.8 (−55.7 to 165.3)	<0.0001	0.458 (0.359 to 0.556)	0.182 (0.103 to 0.291)
Vigorous *vs.* Vigorous (min.day^−1^)	2.6±4.8	10.4±9.4	7.8 (−10.7 to 26.4)	<0.0001	0.259 (0.146 to 0.360)	0.134 (0.092 to 0.339)
MVPA *vs.* MVPA^a^ (MET.min.day^−1^)	155.9±117.9	397.8±248.3	241.9 (−177.6 to 661.5)	<0.0001	0.508 (0.412 to 0.603)	0.222 (0.126 to 0.367)

N = 317,

a moderate and MVPA domains for IPAQ include walking.

b *p*-value for comparison between accelerometer and IPAQ mean values.

Limits of Agreement expressed as the mean difference between methods ±1.96×SD. r = Pearson correlation coefficient; P_c_ = concordance correlation coefficient.

Table 3 shows the ranges for each quartile of sitting/sedentary time (in min.day^−1^) and MVPA (in MET.min.day^−1^) for accelerometer-derived and IPAQ-reported activity measures. Notably, there was no overlap in quartile threshold values for accelerometer-derived and IPAQ-reported MVPA: the threshold for the highest quartile (quartile 4) for accelerometer-derived MVPA fell within the range of the lowest quartile (quartile 1) for IPAQ-reported MVPA.

**Table 3:** Quartile ranges for accelerometer-derived and IPAQ-estimates indices of physical activity and sedentary behaviour.

		Quartile 1	Quartile 2	Quartile 3	Quartile 4
**Sitting/sedentary time (min.day^−1^)**	Accelerometer	<447	447–523	524–578	>578
	IPAQ	<365	365–450	451–535	>535
**MVPA (MET.min.day^−1^)**	Accelerometer	<76	76–125	126–201	>201
	IPAQ	<233	233–325	326–484	>484

### Strength of relationships between IPAQ-reported and accelerometer-derived activity measures and metabolic and vascular disease risk factors

**Figure 2:**
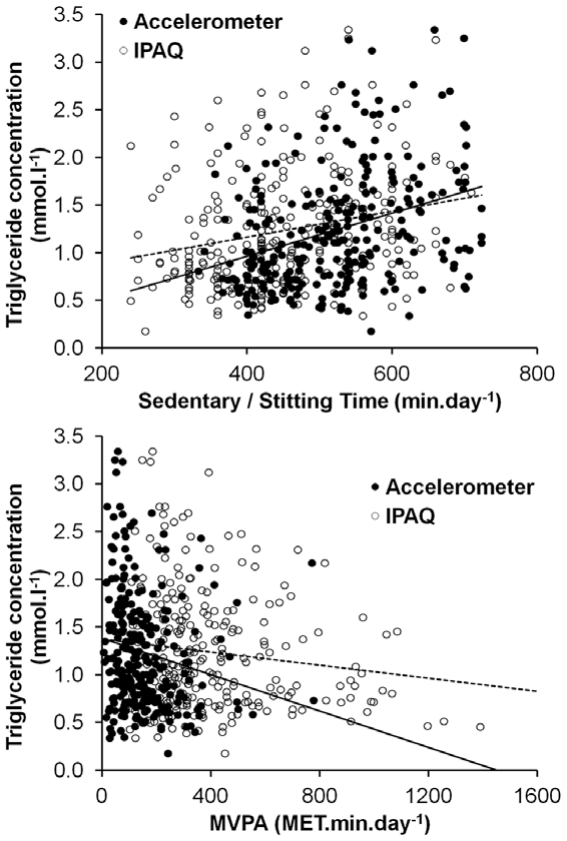
Relationships between sedentary/sitting time and triglyceride concentration (top panel) and MVPA and triglyceride concentration (bottom panel) for accelerometer-derived and IPAQ-reported activity measures. Solid line on each plot represents the linear regression line for accelerometer-derived activity measure; dotted line is the regression line for IPAQ-reported activity measure. Unadjusted data values presented. See Tables 4 and 5 for β coefficients and *p*-values for regression lines, adjusted for age, sex, ethnicity, environment and socio-economic status and smoking.

Table 4 shows β coefficients for the change in metabolic and vascular risk factor levels per 100-minute change in accelerometer-derived sedentary or IPAQ-reported sitting time. Figure 2 provides a graphical representation of the sitting/sedentary time *vs* risk factor relationship for the accelerometer-derived and IPAQ-reported measures, using triglyceride concentration as an illustrative example of the data presented in Table 4. Data and regression lines presented in Figure 2 are for unadjusted data, but β coefficients in Table 4 were adjusted as described in the data analysis section above. For both accelerometer-derived and IPAQ-reported measures, significant positive (negative for HDL cholesterol) β coefficients were observed for all risk factors; in other words increases in sedentary or sitting time were associated with increases in risk factor (or decreases in protective factor) level. For both insulin concentration and HOMA_IR_ there were significant interactions between the linear regression lines for accelerometer-derived *vs* IPAQ-reported relationships: β coefficients were 54% and 67% larger, for insulin and HOMA_IR_ respectively, for the accelerometer-derived compared to IPAQ-reported measure of sedentary behaviour (*p* for both interactions <0.0001). The β coefficients did not differ significantly between accelerometer-derived *vs* IPAQ-reported sitting/sedentary time measures for any other risk factor.

**Table 4:** Regression coefficients (β) for relationships between Actigraph-derived sedentary time and IPAQ-reported sitting time and vascular and metabolic risk factors.

Outcome	Sedentary Time – Accelerometer		Sitting Time – IPAQ		
	β (95%CI)	*p*-value	β (95%CI)	*p*-value	*p*-interaction
Glucose (mmol.l^−1^)	0.2 (0.08 to 0.4)	**0.003**	0.1 (0.01 to 0.002)	**0.008**	0.936
Insulin (mU.l^−1^)	6.8 (5.1 to 8.4)	**<0.0001**	4.4 (3.0 to 5.8)	**<0.0001**	**<0.0001**
HOMA_IR_	1.5 (1.2 to 1.8)	**<0.0001**	0.9 (0.7 to 1.3)	**<0.0001**	**<0.0001**
Total Cholesterol (mmol.l^−1^)	0.31 (0.14 to 0.46)	**<0.0001**	0.21 (0.10 to 0.34)	**0.002**	0.943
HDL cholesterol (mmol.l^−1^)	−0.13 (−0.18 to −0.08)	**<0.0001**	−0.11 (−0.15 to −0.06)	**<0.0001**	0.794
LDL cholesterol (mmol.l^−1^)	0.36 (0.18 to 0.52)	**<0.0001**	0.27 (0.13 to 0.42)	**<0.0001**	0.910
Triglyceride (mmol.l^−1^)	0.17 (0.09 to 0.25)	**<0.0001**	0.09 (0.02 to 0.16)	**0.009**	0.564
Systolic BP (mm Hg)	2.9 (0.8 to 5.0)	**0.006**	2.2 (0.5 to 4.0)	**0.012**	0.445
Diastolic BP (mm Hg)	2.1 (0.4 to 3.7)	**0.012**	1.8 (0.4 to 3.2)	**0.009**	0.100
Body Mass Index (m.kg^−2^)	1.4 (0.8 to 2.1)	**<0.0001**	0.7 (0.2 to 1.3)	**0.005**	0.796
Waist Circumference (cm)	3.1 (1.3 to 4.9)	**0.001**	1.5 (0.02 to 3.0)	**0.046**	0.547
Body Fat (%)	1.8 (1.1 to 0.025)	**<0.0001**	0.6 (0.06 to 1.2)	**0.030**	0.475

N = 317. Data presented as β coefficient and 95%CI for change in risk factor per 100-minute change in sedentary/sitting time, adjusted for age, sex, ethnicity, environment and socio-economic status and smoking. *P*-values values are given for β values for each measurement method and for the interaction between regression coefficients derived from accelerometer *vs* IPAQ measurements.

Table 5 presents the same data for accelerometer-derived and IPAQ-reported measures of MVPA. Figure 2 shows the MVPA *vs* triglyceride concentration relationships for accelerometer-derived and IPAQ-reported measures. For the accelerometer-derived measure of MVPA, significant negative β coefficients (positive for HDL cholesterol) were evident for all risk factor measures except blood pressure, demonstrating that increasing MVPA was associated with reductions in metabolic and vascular risk. However, for the IPAQ-reported measure of MVPA, significant negative β coefficients were only evident for HOMA_IR_, glucose and insulin concentrations. There were significant interactions between the linear regression lines for accelerometer-derived *vs* IPAQ-reported MVPA relationships for insulin concentration, triglyceride concentration and HOMA_IR_, with β coefficients being 43%, 50% and 50% greater for the accelerometer-derived compared to IPAQ-reported MVPA measures, respectively. The β coefficients did not differ significantly between accelerometer-derived *vs* IPAQ-reported MVPA measures for any other risk factor.

**Table 5:** Regression coefficients (β) for relationships between Actigraph-derived and IPAQ-reported MVPA and vascular and metabolic risk factors.

Outcome	MVPA – Accelerometer		MVPA – IPAQ		
	β (95%CI)	*p*	β (95%CI)	*p*	*p*-interaction
Glucose (mmol.l^−1^)	−0.08 (−0.2 to −0.04)	**0.021**	−0.1 (−0.2 to −0.01)	**0.037**	0.305
Insulin (mU.l^−1^)	−2.8 (−4.2 to −1.4)	**<0.0001**	−1.9 (−3.4 to −0.5)	**0.010**	**0.002**
HOMA_IR_	−0.6 (−0.8 to 0.3)	**<0.0001**	−0.4 (−0.7 to −0.2)	**0.002**	**<0.0001**
Total Cholesterol (mmol.l^−1^)	−0.11 (−0.23 to −0.02)	**0.011**	−0.06 (−0.19 to −0.07)	0.388	0.102
HDL cholesterol (mmol.l^−1^)	0.07 (0.03 to 0.11)	**0.001**	0.02 (−0.21 to 0.07)	0.311	0.261
LDL cholesterol (mmol.l^−1^)	−0.14 (−0.27 to −0.01)	**0.042**	−0.06 (−0.20 to 0.08)	0.403	0.181
Triglyceride (mmol.l^−1^)	−0.07 (−0.13 to −0.02)	**0.035**	−0.04 (−0.11 to 0.02)	0.188	**0.011**
Systolic BP (mm Hg)	−1.4 (−3.1 to 0.1)	0.070	−1.6 (−3.2 to 0.04)	0.056	0.084
Diastolic BP (mm Hg)	−0.1 (−1.3 to 1.1)	0.887	0.1 (−1.2 to 1.4)	0.870	0.061
Body Mass Index (m.kg^−2^)	−0.2 (−0.7 to −0.1)	**0.023**	−0.3 (−0.8 to 0.2)	0.188	0.448
Waist Circumference (cm)	−0.6 (−2.0 to −0.7)	**0.036**	−1.1 (−2.5 to 0.3)	0.130	0.258
Body Fat (%)	−1.5 (−1.6 to −0.5)	**<0.0001**	−0.5 (−1.1 to 0.01)	0.055	0.138

N = 317. Data presented as β coefficient and 95%CI for change in risk factor per 100-MET.min change in MVPA, adjusted for age, sex, ethnicity, environment and socio-economic status and smoking. *P*-values values are given for β values for each measurement method and for the interaction between regression coefficients derived from accelerometer *vs* IPAQ measurements.

### Trends in metabolic and vascular disease risk factors across quartiles for IPAQ-reported and accelerometer-derived measures

**Figure 3:**
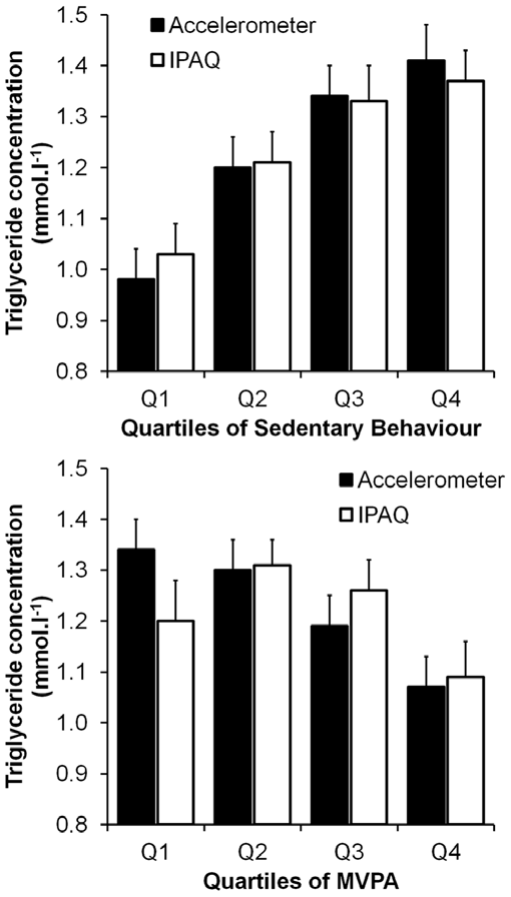
Triglyceride concentrations by quartile of sitting/sedentary time (top panel) or MVPA (bottom panel) for the accelerometer-derived and IPAQ-reported activity measures. Trends for triglyceride concentration by sitting/sedentary time quartile significant for both accelerometer (*p* = 0.0001) and IPAQ (*p* = 0.014) measures. Trends for triglyceride concentration by MVPA quartile significant for the accelerometer (*p* = 0.022), but not the IPAQ (*p* = 0.139), activity measure.

Table 6 shows risk factor levels for each quartile of accelerometer-derived sedentary time or IPAQ-reported sitting time. As an illustrative example, the data for triglyceride concentrations by quartile of sitting/sedentary time for the accelerometer and IPAQ measures are shown in Figure 3. When the data were analysed in this manner, significant trends to increase risk factor values with increasing time spent in sedentary behaviours were observed for insulin, HOMA_IR_, triglyceride, BMI and body fat, for both accelerometer-derived and IPAQ-reported measures. A significant negative trend for HDL with increasing sedentary behaviour was evident for both measures. For total and LDL cholesterol, systolic blood pressure and waist circumference significant trends were observed for accelerometer-derived but not IPAQ-reported measures; in other words increases in objectively measured sedentary behaviours but not sitting time were associated with increases in these risk factor levels. No significant trends were found for glucose or diastolic blood pressure for either the accelerometer-derived and IPAQ-reported measures of sedentary behaviour. Further adjustment for MVPA in the models did not alter the significance of any of these findings. There were no significant interactions between the accelerometer-derived sedentary time and IPAQ-reported sitting time *vs* risk factor trends for any risk factor.

**Table 6:** Vascular and metabolic risk factor values by quartile of accelerometer-derived sedentary time and IPAQ-reported sitting time.

Risk Factor	Activity Measurement Method	Quartile 1 (n = 79)	Quartile 2 (n = 79)	Quartile 3 (n = 79)	Quartile 4 (n = 80)	*p*-trend*	*p*-trend#
Glucose (mmol.l^−1^)	Accelerometer	5.34±0.12	5.45±0.13	5.52±0.12	5.77±0.13	0.200	0.358
	IPAQ	5.44±0.13	5.33±0.12	5.46±0.14	5.84±0.12	0.058	0.054
Insulin (mU.l^−1^)	Accelerometer	2.82±1.22	6.10±1.26	9.11±1.22	15.9±1.29	**0.0001**	**0.0001**
	IPAQ	3.78±1.23	5.76±1.16	9.56±1.33	14.92±1.24	**0.0001**	**0.0001**
HOMA_IR_	Accelerometer	0.70±0.27	1.52±0.28	2.21±0.27	4.05±0.28	**0.0001**	**0.0001**
	IPAQ	0.94±0.27	1.41±0.25	2.32±0.29	3.81±0.27	**0.0001**	**0.0001**
Total Cholesterol (mmol.l^−1^)	Accelerometer	4.41±0.13	4.60±0.14	4.99±0.13	5.02±0.13	**0.004**	**0.019**
	IPAQ	4.55±0.13	4.69±0.12	4.81±0.14	4.97±0.13	0.381	0.322
HDL cholesterol (mmol.l^−1^)	Accelerometer	1.07±0.04	1.01±0.04	0.83±0.04	0.74±0.04	**0.0001**	**0.0001**
	IPAQ	1.10±0.04	0.94±0.03	0.82±0.04	0.78±0.04	**0.0001**	**0.0001**
LDL cholesterol (mmol.l^−1^)	Accelerometer	2.89±0.13	3.04±0.14	3.53±0.13	3.63±0.14	**0.0009**	**0.005**
	IPAQ	2.98±0.14	3.19±0.13	3.38±0.15	3.56±0.14	0.125	0.115
Triglyceride (mmol.l^−1^)	Accelerometer	0.98±0.06	1.20±0.06	1.34±0.06	1.41±0.07	**0.0001**	**0.002**
	IPAQ	1.03±0.06	1.21±0.06	1.33±0.07	1.37±0.06	**0.014**	**0.013**
Systolic BP (mm Hg)	Accelerometer	120.7±1.6	120.2±1.5	126.3±1.6	126.0±1.6	**0.013**	**0.044**
	IPAQ	121.2±1.6	121.5±1.5	124.6±1.7	126.1±1.6	0.187	0.191
Diastolic BP (mm Hg)	Accelerometer	74.3±1.29	74.9±1.28	78.3±1.32	78.5±1.31	0.069	0.088
	IPAQ	74.7±1.30	75.6±1.22	78.6±1.36	77.4±1.29	0.285	0.278
Body Mass Index (kg.m^−2^)	Accelerometer	27.5±0.51	28.5±0.51	30.2±0.53	30.6±0.52	**0.0001**	**0.0003**
	IPAQ	27.7±0.52	29.3±0.48	29.6±0.54	30.2±0.51	**0.047**	**0.049**
Waist circumference (cm)	Accelerometer	98.8±1.46	100.1±1.45	104.1±1.49	106.3±1.48	**0.003**	**0.003**
	IPAQ	98.4±1.47	102.5±1.38	104.3±1.54	104.1±1.46	0.066	0.065
Body Fat (%)	Accelerometer	28.5±0.58	29.4±0.57	30.8±0.59	32.6±0.59	**0.0001**	**0.007**
	IPAQ	28.7±0.58	30.1±0.54	31.5±0.61	31.3±0.58	**0.018**	**0.019**

Values are means ± SEM for each quartile.

* Model adjusted for age, ethnicity, sex, environment, socio-economic status and smoking status.

# Model adjusted for age, ethnicity, sex, environment, socio-economic status, smoking status and MVPA.

Table 7 presents the same data for accelerometer-derived and IPAQ-reported measures of MVPA. Data for triglyceride concentration by quartile of MVPA for the accelerometer and IPAQ measures are also shown in Figure 3. For the accelerometer-derived measure of MVPA, significant negative trends in risk factor levels with increasing MVPA were evident for insulin, HOMA_IR_, triglyceride, systolic blood pressure and percentage body fat and a positive trend was observed for HDL cholesterol. In other words, increasing MVPA was associated with reductions in metabolic and vascular risk. However, after adjusting the model for sedentary time, only the trends for insulin and HOMA_IR_ remained statistically significant.

**Table 7:** Vascular and metabolic risk factors values by quartile of accelerometer-derived and IPAQ-reported time spent in MVPA.

Risk Factor	Activity Measurement Method	Quartile 1 (n = 79)	Quartile 2 (n = 79)	Quartile 3 (n = 79)	Quartile 4 (n = 80)	*p*-trend*	*p*-trend#
Glucose (mmol.l^−1^)	Accelerometer	5.58±0.12	5.66±0.12	5.46±0.12	5.35±0.13	0.348	0.723
	IPAQ	5.63±0.16	5.67±0.10	5.41±0.12	5.30±0.13	0.153	0.164
Insulin (mU.l^−1^)	Accelerometer	14.93±1.27	6.87±1.25	6.82±1.27	4.26±1.32	**0.0001**	**0.0005**
	IPAQ	13.79±1.62	7.63±1.06	6.83±1.28	7.08±1.36	**0.015**	0.215
HOMA_IR_	Accelerometer	3.67±0.29	1.81±0.28	1.68±0.28	1.05±0.29	**0.0001**	**0.0002**
	IPAQ	3.51±0.36	1.95±0.24	1.67±0.28	1.69±0.31	**0.002**	0.093
Total Cholesterol (mmol.l^−1^)	Accelerometer	4.88±0.13	4.88±0.13	4.60±0.13	4.62±0.13	0.288	0.818
	IPAQ	4.68±0.16	4.98±0.11	4.70±0.13	4.49±0.14	0.055	**0.047**
HDL cholesterol (mmol.l^−1^)	Accelerometer	0.82±0.04	0.89±0.04	0.95±0.04	1.02±0.04	**0.012**	0.757
	IPAQ	0.89±0.05	0.89±0.03	0.90±0.04	0.99±0.04	0.413	0.505
LDL cholesterol (mmol.l^−1^)	Accelerometer	3.45±0.14	3.40±0.14	3.11±0.14	3.11±0.14	0.218	0.847
	IPAQ	3.24±0.18	3.49±0.11	3.22±0.14	2.99±0.15	0.092	0.085
Triglyceride (mmol.l^−1^)	Accelerometer	1.34±0.06	1.30±0.06	1.19±0.06	1.07±0.06	**0.022**	0.586
	IPAQ	1.20±0.08	1.31±0.05	1.26±0.06	1.09±0.07	0.139	0.128
Systolic BP (mm Hg)	Accelerometer	126.8±1.6	123.1±1.5	119.6±1.6	123.5±1.6	**0.019**	0.064
	IPAQ	124.2±1.9	124.2±1.4	122.2±1.5	122.3±1.6	0.707	0.709
Diastolic BP (mm Hg)	Accelerometer	78.6±1.2	75.5±1.2	74.5±1.3	77.1±1.3	0.134	0.168
	IPAQ	77.9±1.5	76.9±1.1	75.3±1.2	76.1±1.3	0.626	0.609
Body Mass Index (kg.m^−2^)	Accelerometer	29.5±0.53	29.5±0.52	29.4±0.53	28.5±0.53	0.486	0.723
	IPAQ	29.4±0.64	29.2±0.47	29.2±0.52	29.1±0.54	0.993	0.994
Waist Circumference (cm)	Accelerometer	102.4±1.4	103.1±1.4	103.6±1.4	100.1±1.5	0.349	0.332
	IPAQ	105.2±1.7	100.5±1.3	101.9±1.4	102.7±1.5	0.199	0.195
Body Fat (%)	Accelerometer	31.8±0.58	30.9±0.57	30.1±0.59	28.4±0.59	**0.0006**	0.083
	IPAQ	30.9±0.71	30.7±0.52	30.3±0.58	29.5±0.60	0.588	0.590

Values are means ± SEM for each quartile.

* Model adjusted for age, ethnicity, sex, environment, socio-economic status and smoking status.

# Model adjusted for age, ethnicity, sex, environment, socio-economic status, smoking status and sedentary/sitting time.

In contrast, for the IPAQ-reported data, significant negative trends in risk factor levels with increasing MVPA were only evident for insulin and HOMA_IR_ concentrations. A borderline (*p* = 0.055) trend towards a decrease in total cholesterol with increasing MVPA was also observed. After adjusting the model for sitting time, the trend to decrease cholesterol with increasing MVPA became statistically significant, but the significant trends for insulin and HOMA_IR_ were lost. To determine whether including walking in the IPAQ-reported MVPA measure influenced these dose-response relationships, the analyses were repeated excluding walking from the IPAQ MVPA measure – i.e. defining MVPA as: minutes of moderate activity (excluding walking)×4.0 METS+minutes of vigorous activity×8.0 METS. This analysis yielded essentially identical results. No significant interactions were observed between the accelerometer-derived and IPAQ-reported MVPA *vs* risk factor trends for any risk factor.

## Discussion

The main findings of this study were: 1) Compared to objective, accelerometer-derived measures, using the IPAQ to determine activity measures led to significant over-reporting of physical activity and under-reporting of sedentary behaviour. The concordance correlation coefficient for accelerometer-derived *vs* IPAQ-reported activity measures was reasonably strong for sedentary behaviour (P_c_ = 0.52, *p*<0.0001), but much weaker for indices of physical activity (P_c_≤0.22 for all measures), indicating that the IPAQ quantified sedentary behaviour more accurately than it quantified physical activity. 2) For some metabolic and vascular disease risk factors, significant trends were observed between amount of sedentary behaviour or MVPA and the risk factor when activity was assessed by IPAQ. However, for other risk factors significant trends with amount of sedentary behaviour or MVPA were only apparent when activity was assessed objectively by accelerometer. In addition, significant interactions were observed for some risk factors (insulin, triglyceride and HOMA_IR_) in the gradient of the activity *vs* risk factor regression lines for accelerometer-derived compared to IPAQ-reported activity measures. These data suggest that, compared to the use of objective accelerometry, using the IPAQ to estimate physical activity and sedentary behaviour may result in failure to detect real relationships with metabolic and vascular disease risk factors or in underestimation of the strength of those relationships. 3) Irrespective of the activity measurement method, time spent engaging in sedentary behaviour was more robustly associated with the cardio-metabolic risk profile than time spent undertaking MVPA.

The IPAQ systematically overestimated vigorous activity by ∼8 minutes per day (4-fold), and, when walking was included in the moderate activity domain, overestimated moderate activity by 55 minutes per day – a 2.6-fold difference. However, agreement between accelerometer- and IPAQ-derived indices of sedentary behaviour was somewhat better with a mean difference of 69 minutes per day (∼13%), despite the measures not being directly equivalent (i.e. time spent sitting down *vs* time at <100 accelerometer counts.min^−1^). These data suggest that while reports of absolute amounts of physical activity determined by the IPAQ questionnaire should be viewed with caution – a minute of IPAQ-reported physical activity is not equivalent to a minute of accelerometer-derived activity – questionnaire-derived estimates of sedentary time agree reasonably well with the objective measure. The Pearson correlations observed between IPAQ-reported and accelerometer-derived indices of physical activity in the present study were in line with, or slightly higher than, previous reports in the literature [14]–[16], indicating that the IPAQ performed at least as well in our hands as for others. Thus, the relative differences in the magnitude of dose-response relationships with risk factors between IPAQ-reported and accelerometer-derived activity measures observed in the present study are likely to be broadly transferable to populations beyond that used in the present study.

In the analysis reported here, the relationships between activity variables and risk factor levels were determined in two different ways. Firstly, β-coefficients for the change in risk factor level per unit change in sedentary behaviour or MVPA were calculated for the IPAQ-reported and accelerometer-determined activity measures. This provides a measure of the unit change in risk factor per 100 minute change in sedentary behaviour or per 100 MET.min^−1^ change (equivalent to 25 minutes of moderate activity) in MVPA. This approach revealed significant relationships between sedentary behaviour and all of the measured risk factors, irrespective of whether sedentary behaviour was assessed by IPAQ or accelerometer. However, the β-coefficients for insulin and HOMA_IR_ were over 50% larger when sedentary behaviour was determined objectively by accelerometer, indicating that using the IPAQ for activity assessment leads to a significant underestimation of the steepness of the dose-response relationship between sedentary behaviour and metabolic risk factors related to insulin resistance. The IPAQ also performed less well for MVPA than it did for sitting time in revealing significant associations with risk factors that were evident when activity was objectively measured. The IPAQ missed significant trends for MVPA that were evident for the accelerometer for total, LDL and HDL cholesterol, triglyceride, BMI, waist circumference and body fat, with only significant trends for glucose, insulin and HOMA_IR_ being detected. Furthermore, the β-coefficients for insulin, triglyceride and HOMA_IR_ were significantly lower for the IPAQ-reported compared to accelerometer-derived MVPA measures. These findings are in broad agreement with the NHANES report which found that the relationships of MVPA with a large number of risk factors were less strong when activity was assessed by questionnaire rather than accelerometer [18]. The present findings extend the NHANES data by presenting data for sedentary behaviour as well as MVPA.

In a separate analysis, the trends for activity measures with risk factors were presented in terms of change in risk factor per quartile change in MVPA or sedentary behaviour within the population, rather than change in risk factor by change in sedentary behaviour or MVPA in terms of absolute units (i.e. per min.day^−1^ or per MET.min^−1^.day^−1^). This approach, in effect, adjusts for any systematic bias in reporting from the questionnaires. For example, the mean reported MET.min.day^−1^ value for MVPA for IPAQ was 2.6 times the accelerometer-derived MVPA measure, but a consistent 2.6 fold over-reporting of MVPA with the IPAQ compared to the accelerometer measure would have no effect on the population distribution into quartiles of MVPA. Using this approach, the present data revealed that the trends for changes in MVPA/sedentary behaviour across the population distribution with a wide range of vascular and metabolic risk factors were broadly similar – there were no significant interactions with measurement method in the activity *vs* risk factor relationships for any risk factor – but not all of the significant trends identified when activity was objectively quantified were observed with the IPAQ. This suggests that imprecision of measurement of activity – i.e. regression dilution bias – when using the IPAQ reduced the ability to detect significant trends with some risk factors, but the overall pattern of the activity *vs* risk factor trends were similar for both measurement approaches. Thus, the relative imprecision of activity measurement by IPAQ could potentially be overcome by studying larger cohorts, but accelerometers offer the advantage of detecting significant trends with risk factors in smaller numbers of individuals, when activity variables are expressed in terms of position in the population distribution. However, while this analytical approach is useful in determining general trends between activity and risk factors, it does have the clear limitations of being unable of providing absolute activity values to quantify the dose-response relationships, as well as having less statistical power than when activity behaviour is considered as a continuous variable.

The observation that the dose-response relationship between activity measures and some risk factors is quantitatively different between self-reported and objectively measured activity is an important consideration when formulating guidelines for physical activity. Current physical activity guidelines, which were largely based on evidence from self-report activity measures, recommend that adults undertake 150 minutes of moderate intensity physical activity or 60–75 minutes of vigorous activity per week to maintain and improve health [10], [11]. The present findings suggest that the amounts of activity required for good health are likely to be lower for objectively measured (compared to reported) activity, an issue which has been alluded to by others [18], [22], [31]. Indeed, accelerometer-derived measures of physical activity are consistently lower than values obtained from self-reported data [22]. Thus, it is important that objective activity monitoring methods are used in the epidemiological studies to determine the optimal activity levels for guidelines as well as in surveillance of activity levels within the population. Using objective measures to assess compliance with guidelines that were based on evidence from self-reported activity, risks providing an inaccurate picture of the proportion of the population who are insufficiently active, which can have important implications with respect to formulation of physical activity policy.

It is of interest that sedentary behaviour was more consistently associated with vascular and metabolic risk factor levels than MVPA was. The difference was most evident for the IPAQ-reported activity measures (12 *vs* 3 significant β-coefficients with risk factors for sitting time *vs* MVPA), so may reflect in part the fact that the IPAQ quantifies sedentary behaviour more accurately than MVPA. However, the effect was still evident, albeit to a lesser extent, for accelerometer-derived measures (12 *vs* 10 significant β-coefficients). Furthermore, adjusting the sedentary behaviour *vs* risk factor trends for MVPA did not alter the strength of association. A number of significant trends for MVPA *vs* risk factor were lost after adjustment for sedentary behaviour, even for accelerometer-derived activity measures, suggesting that this is a real biological effect and not simply a consequence of measurement error. This observation is in agreement with other published data revealing a larger effect of sedentary behaviour than physical activity on a number of vascular and metabolic risk factors [3], [32], although this is not an unequivocal finding [33]. Nevertheless, the present findings add to the growing literature highlighting the important influence of sedentary behaviour on indices of vascular and metabolic health.

A particular strength of this study is that the study population was diverse. Participants were men and women, with a wide range of educational and socioeconomic backgrounds and spanned a wide range of activity levels. Mean activity levels in the present cohort were higher than in NHANES, which represented a nationally representative sample for the US, and half the cohort lived in rural settings. Our findings were robust to adjustment for age, sex, ethnicity, environment and socio-economic status, and thus should be generalisable across a wide demographic range and particularly to populations outside the US where physical activity levels may be higher. This is also the first study to compare the effects of objective *vs* subjective measurements of sedentary time on the dose-response relationship with vascular and metabolic risk factors. However, while we showed good agreement between the accelerometer-derived and IPAQ-reported sedentary time measures, it is important to recognise the <100 accelerometer counts per minute is not exactly the same as time spent sitting down, as the former measure can also include standing and some very low intensity upright activities (such as swaying). It has been suggested that contractions in postural muscles elicited by standing may confer some metabolic benefit compared to sitting [34], thus the potential inclusion of standing activities in our accelerometer-derived sedentary behaviour measure could conceivably have attenuated the apparent risk factor dose-response relationships. Further study, using inclinometers, comparing the risk factor dose-response relationships for objectively- and questionnaire-assessed sitting time is needed to address this issue.

In conclusion, the findings of this study indicate that using IPAQ to determine physical activity or sitting time reveals a number of significant trends with metabolic and vascular disease risk factors. However, the IPAQ missed some significant trends that were evident when activity was objectively assessed, particularly for MVPA, and led to underestimation of the strength of some relationships between activity and risk factors. Thus, a degree of caution is advised when using activity measurements obtained from the IPAQ to quantify dose-response relationships for activity and risk factors for metabolic and vascular disease.

## References

[1] Gill JM, Cooper AR (2008). Physical activity and prevention of type 2 diabetes mellitus.. Sports Med.

[2] Nocon M, Hiemann T, Muller-Riemenschneider F, Thalau F, Roll S (2008). Association of physical activity with all-cause and cardiovascular mortality: a systematic review and meta-analysis.. Eur J Cardiovasc Prev Rehabil.

[3] Gill JM, Bhopal R, Douglas A, Wallia S, Bhopal R (2011). Sitting Time and Waist Circumference Are Associated With Glycemia in U.K. South Asians: Data from 1,228 adults screened for the PODOSA trial.. Diabetes Care.

[4] Katzmarzyk PT, Church TS, Craig CL, Bouchard C (2009). Sitting time and mortality from all causes, cardiovascular disease, and cancer.. Med Sci Sports Exerc.

[5] Dunstan DW, Barr EL, Healy GN, Salmon J, Shaw JE (2010). Television viewing time and mortality: the Australian Diabetes, Obesity and Lifestyle Study (AusDiab).. Circulation.

[6] Hu FB, Leitzmann MF, Stampfer MJ, Colditz GA, Willett WC (2001). Physical activity and television watching in relation to risk for type 2 diabetes mellitus in men.. Arch Intern Med.

[7] Hu FB, Li TY, Colditz GA, Willett WC, Manson JE (2003). Television watching and other sedentary behaviors in relation to risk of obesity and type 2 diabetes mellitus in women.. JAMA.

[8] Terwee CB, Mokkink LB, van Poppel MN, Chinapaw MJ, van MW (2010). Qualitative attributes and measurement properties of physical activity questionnaires: a checklist.. Sports Med.

[9] Lagerros YT, Lagiou P (2007). Assessment of physical activity and energy expenditure in epidemiological research of chronic diseases.. Eur J Epidemiol.

[10] Haskell WL, Lee IM, Pate RR, Powell KE, Blair SN (2007). Physical activity and public health: updated recommendation for adults from the American College of Sports Medicine and the American Heart Association.. Med Sci Sports Exerc.

[11] Department of Health (2011). Start Active, Stay Active: a report on physical activity for health from the four home countries'. Chief Medical Officers.

[12] Shephard RJ (2003). Limits to the measurement of habitual physical activity by questionnaires.. Br J Sports Med.

[13] van Poppel MN, Chinapaw MJ, Mokkink LB, van MW, Terwee CB (2010). Physical activity questionnaires for adults: a systematic review of measurement properties.. Sports Med.

[14] Craig CL, Marshall AL, Sjostrom M, Bauman AE, Booth ML (2003). International physical activity questionnaire: 12-country reliability and validity.. Med Sci Sports Exerc.

[15] Hagstromer M, Oja P, Sjostrom M (2006). The International Physical Activity Questionnaire (IPAQ): a study of concurrent and construct validity.. Public Health Nutr.

[16] Rosenberg DE, Bull FC, Marshall AL, Sallis JF, Bauman AE (2008). Assessment of sedentary behavior with the International Physical Activity Questionnaire.. J Phys Act Health.

[17] Frost C, Thompson SG (2000). Correcting for regression dilution bias: comparison of methods for a single predictor variable.. J R Statist Soc A.

[18] Atienza AA, Moser RP, Perna F, Dodd K, Ballard-Barbash R (2011). Self-reported and objectively measured activity related to biomarkers using NHANES.. Med Sci Sports Exerc.

[19] Fan AZ, Ham SA, Muppidi SR, Mokdad AH (2009). Validation of reported physical activity for cholesterol control using two different physical activity instruments.. Vasc Health Risk Manag.

[20] Celis-Morales CA, Perez-Bravo F, Ibanes L, Sanzana R, Hormazabal E (2011). Insulin resistance in Chileans of European and Indigenous descent: evidence for an ethnicity x environment interaction.. PLoS ONE.

[21] Freedson PS, Melanson E, Sirard J (1998). Calibration of the Computer Science and Applications, Inc. accelerometer.. Med Sci Sports Exerc.

[22] Troiano RP, Berrigan D, Dodd KW, Masse LC, Tilert T (2008). Physical activity in the United States measured by accelerometer.. Med Sci Sports Exerc.

[23] Hagstromer M, Oja P, Sjostrom M (2007). Physical activity and inactivity in an adult population assessed by accelerometry.. Med Sci Sports Exerc.

[24] Marfell-Jones M, Olds T, Stewart A, Carter L (2006). International standards for anthropometric assessment.

[25] Durnin JVGA, Womersley J (1974). Body fat assessed from total body density and its estimation from skinfold thickness: measurements on 481 men and women aged from 16 to 72 years.. British Journal of Nutrition.

[26] El Assaad MA, Topouchian JA, Asmar RG (2003). Evaluation of two devices for self-measurement of blood pressure according to the international protocol: the Omron M5-I and the Omron 705IT.. Blood Press Monit.

[27] Friedewald WT, Levy RI, Fredrickson DS (1972). Estimation of the concentration of low-density lipoprotein cholesterol in plasma, without use of the preparative ultracentrifuge.. Clin Chem.

[28] Hoffmeyer-Zlotnik JHP, Wolf C, ESOMAR (1997). The ESOMAR Standard Demographic Classification. A System of International Socio-Economic Classification of Respondents to Survey Research.. Advances in Cross-National Comparison. A European Working Book for Demographic and Socio-Economic Variables.

[29] National Socioeconomic Survey (2006). Poll of Quality of Life in Households.

[30] Bland JM, Altman DG (1999). Measuring agreement in method comparison studies.. Stat Methods Med Res.

[31] Troiano RP (2007). Large-scale applications of accelerometers: new frontiers and new questions.. Med Sci Sports Exerc.

[32] Healy GN, Wijndaele K, Dunstan DW, Shaw JE, Salmon J (2008). Objectively measured sedentary time, physical activity, and metabolic risk: the Australian Diabetes, Obesity and Lifestyle Study (AusDiab).. Diabetes Care.

[33] Ekelund U, Brage S, Griffin SJ, Wareham NJ (2009). Objectively measured moderate- and vigorous-intensity physical activity but not sedentary time predicts insulin resistance in high-risk individuals.. Diabetes Care.

[34] Hamilton MT, Hamilton DG, Zderic TW (2007). Role of low energy expenditure and sitting in obesity, metabolic syndrome, type 2 diabetes, and cardiovascular disease.. Diabetes.

